# Sensitivity and Frequency-Response Improvement of a Thermal Convection–Based Accelerometer

**DOI:** 10.3390/s17081765

**Published:** 2017-08-02

**Authors:** Maeum Han, Jae Keon Kim, Jin-Hyoung Park, Woojin Kim, Shin-Won Kang, Seong Ho Kong, Daewoong Jung

**Affiliations:** 1 School of Electronics Engineering, College of IT Engineering, Kyungpook National University, Daegu 41566, Korea; mehan@knu.ac.kr (M.H.); swkang@knu.ac.kr (S.-W.K.); 2 Construction Equipment R&D Group, Korea Institute of Industrial Technology (KITECH), Daegu 42994, Korea; jh.park@kitech.re.kr; 3 Department of Sensor and Display Engineering, Kyungpook National University, Daegu 41566, Korea; kjg@kitech.re.kr; 4 Aircraft System Technology Group, Korea Institute of Industrial Technology (KITECH), Daegu 42994, Korea; 5 Mechatronics Technology Convergence R&D Group, Korea Institute of Industrial Technology (KITECH), Daegu 42994, Korea; woojinkim@kitech.re.kr

**Keywords:** accelerometer, frequency, acceleration, heat convection

## Abstract

This paper presents a thermal convection–based sensor fabricated using simple microelectromechanical systems (MEMS)-based processes. This sensor can be applied to both acceleration and inclination measurements without modifying the structure. Because the operating mechanism of the accelerometer is the thermal convection of a gas medium, a simple model is proposed and developed in which the performance of the thermal convection–based accelerometer is closely associated with the Grashof number, G_r_ and the Prandtl number, P_r_. This paper discusses the experiments that were performed by varying several parameters such as the heating power, cavity size, gas media, and air pressure. The experimental results demonstrate that an increase in the heating power, pressure, and cavity size leads to an increase in the accelerometer sensitivity. However, an increase in the pressure and/or cavity size results in a decrease in the frequency bandwidth. This paper also discusses the fact that a working-gas medium with a large thermal diffusivity and small kinematic viscosity can widen the frequency bandwidth and increase the sensitivity, respectively.

## 1. Introduction

Microelectromechanical systems (MEMS)-based devices integrate various mechanical elements, sensors, actuators, and electronics onto a silicon substrate to achieve a multitude of different tasks over a diverse range of fields. MEMS-based sensors are increasingly being used for commercial and industrial applications, such as pressure sensors, gas sensors, RF (Radio frequency) switches, and flow sensors. One of the most important MEMS devices is the accelerometer. Many types of accelerometers used to measure vibration, shock, and inertial motion have been developed for application in various industrial fields. Most conventional accelerometers detect the acceleration using a solid proof mass and are based on the changes in the capacitance [1,2] or the piezo effect [3,4]. However, MEMS-based accelerometers based on the proof-mass system suffer from problems such as electromagnetic interference (EMI), the effect of parasitic electrostatic force, and fabrication complexity [5,6,7,8,9]. To overcome these problems, a thermal type of MEMS-based accelerometer that does not require a solid proof mass has been developed and fabricated, as described in previous papers [5,6,7,8,9,10,11,12]. The key features of these accelerometers are that they have no solid proof mass, and their operation is based on air convection through a thermal exchange with a microheater in an enclosed chamber. The researchers found that gas is a good working medium compared with solid or liquid medium because no mechanical parts are present and they are anticorrosive and stable in the atmosphere.

Our group previously reported a thermal convection–based sensor for tilting measurement [13]. The sensor can be applied to both acceleration and inclination measurements without modifying the structure. The current paper presents the experiments carried out to examine the parameters that affect the sensitivity and frequency characteristics of thermal convective accelerometers. Special efforts were made considering improvements to the frequency response of the accelerometer because currently available reports on experimental studies concerning the frequency response are few [14]. To improve the sensitivity and frequency response, the current study concentrated on optimizing these different parameters: the heating power, operating frequency, gas medium, operating pressure, and cavity volume of an encapsulated microchamber. In addition, the proposed thermal convection–based accelerometer response was theoretically evaluated, and its operating characteristics were measured through experimentation. Various simple models were used to better understand the sensitivity and frequency response of the accelerometer. The more influential parameters were then taken into account in the sensor design.

## 2. Materials and Methods 

### 2.1. Device Structure

**Figure 1:**
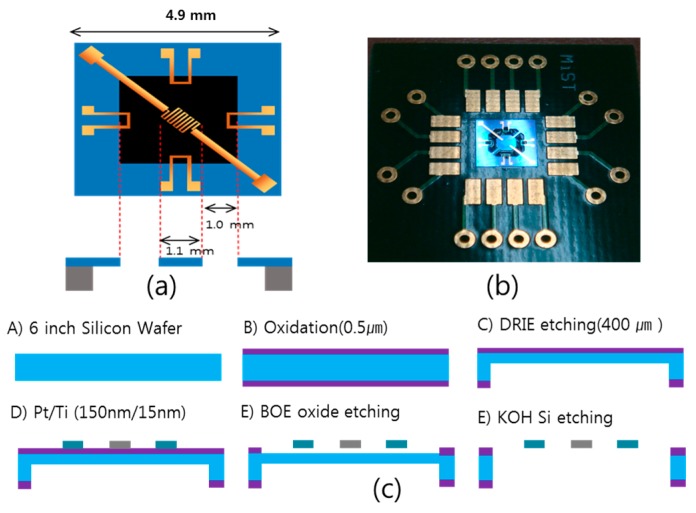
(**a**) Schematic view, (**b**) fabricated sensor on the PCB board and (**c**) fabrication process of the thermal convection–based accelerometer.

Figure 1a,b show the structure of the thermal convection–based accelerometer. It consists of a central microheater to heat the gas medium in the cavity, and four temperature sensors located from either sides of the heater where the gas medium flows across the temperature sensors through free convection. Figure 1c illustrates the fabrication process of the sensor. The silicon (Si) substrate was prepared with the 0.5 μm thickness of silicon dioxide layer. The back side of the Si wafer was etched and the heater and temperature sensors at the bottom wafer are made of platinum (Pt), which has very stable physical properties and operates with a high temperature coefficient of resistance of 3.93 × 10^–3^ (°C)^−1^. The frond side of Si was removed by wet etching to form thermal isolation in the microchamber. Finally, the sensor is packaged in a sealed microchamber that contains a working medium, e.g., air or nitrogen (N_2_).

### 2.2. Principles of Operation

**Figure 2:**
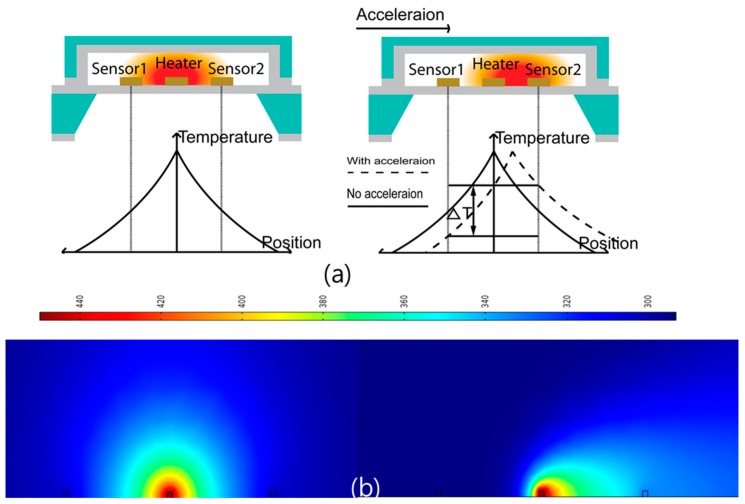
The proposed convective sensor (**a**) basic principle and (**b**) simulation analysis (1 g at 450 K and atmospheric pressure).

Figure 2a shows the temperature distribution in the cavity when the accelerometer is not subjected to acceleration. When power is introduced to the heater, the temperature around the heater rises, creating thermal convection in the cavity. In this case, the temperature profile of the gas medium in the cavity is symmetrically sensed by both temperature sensors, as shown by the straight line. No temperature difference is detected between the two temperature sensors; thus, identical electrical resistances and/or voltages are produced. When the accelerometer is subjected to acceleration, the temperature profile shifts along the direction of the applied acceleration, as shown Figure 2b. This condition results in an increase in temperature around one temperature sensor and a decrease in temperature around the other sensor, generating a temperature difference between the two temperature sensors that is proportional to the magnitude of the applied acceleration. By measuring the electrical signal produced from the temperature difference, the acceleration can be determined.

### 2.3. Theory of Operation

Because the working mechanism of the convective accelerometer is based on thermal convection, a method to improve its performance is to provide an appropriate convection environment to the accelerometer. Generally, two non-dimensional parameters, namely the Grashof number G_r_ and the Prandtl number P_r_, are introduced to investigate the effect of thermal convection [15].

(1) $$G_{r}=\frac{g\rho^{2}\beta L^{3}\Delta T}{\mu^{2}}$$

(2) $$P_{r}=\frac{\mu }{\alpha }$$

Here g, ρ, β, L, ΔT, μ, and α are the applied acceleration, gas density, coefficient of volumetric expansion, characteristic size (generally denotes the cavity size), temperature difference between the heater and boundary of the sensor, kinematic viscosity, and thermal diffusivity, respectively. As expressed in Equations (1) and (2), these parameters can be used to predict the effects of the working-fluid properties on the sensitivity and frequency response of the thermal convection-based accelerometer.

Previous works have shown that the temperature difference in the thermal convection-based sensor is linearly proportional to the G_r_ number; thus, a larger G_r_ number results in higher sensitivity of the thermal convection-based accelerometer for a given acceleration [10,11,12]. Therefore, increasing the value of the Gr number is essential to obtain good sensitivity of the thermal accelerometer.

According to Equation (1), the following design parameters can be considered to increase the sensitivity of the thermal accelerometer:

(i) Large heating power and lower ambient temperature, which corresponds to increasing T,
(ii) Increase in the characteristic size, which corresponds to increasing L,
(iii) Selection of a gas medium with large gas density ρ and small kinematic viscosity μ.

Previous papers on thermal accelerometer have discussed the dependence of performance on G_r_ and P_r_, important factors in sensitivity and frequency response [11,12,13,14,16,17]. In particular, the frequency response of a thermal accelerometer can therefore be improved by taking into account the thermal variations in the gas properties and the dependence on the air thermal conductivity [12]. To obtain a fast and wider frequency response, we need to highly influence the thermal physical properties of the gas medium. A large thermal conductivity (α) and a small gas density (ρ) will accelerate the thermal diffusion, which consequently facilitates the heat balance in the cavity of the accelerometer and provides a fast frequency response to the accelerometer.

In addition, the frequency response is associated with the cavity dimensions and the distance between the heater and temperature sensor. We can deduce that achieving heat uniformity in a larger cavity of an accelerometer at given heater power takes a longer time and shows a slower frequency response. On the other hand, the gas medium around the heater can quickly reach the temperature sensor when the distance between the heater and temperature sensor is small.

All the above-mentioned methods to improve the sensitivity and frequency response of the thermal convection-based accelerometer must be experimentally carried out based on G_r_ and P_r_ parameters to optimize the sensor structural design and maximize the performance of the accelerometer.

## 3. Results and Discussion

### 3.1. Effects of the Heating Power

**Figure 3:**
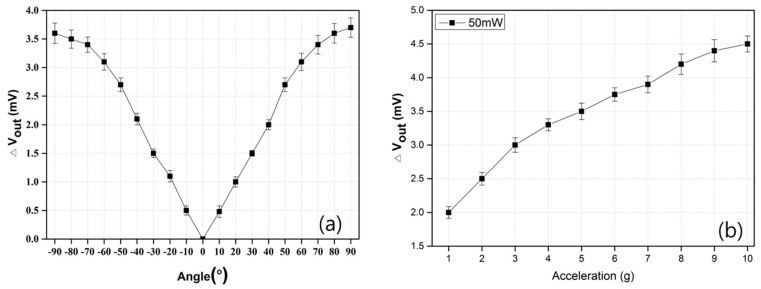
The output voltage of the accelerometer as a function of (**a**) tilting angles and (**b**) accelerations.

The sensitivities of the thermal accelerometer were measured by two methods that rely on field applications. One method that measures the sensitivity, which is called static sensitivity, was undertaken from −90° (−g) to +90° (+g) by rotating the accelerometer under standard conditions (room temperature and atmospheric pressure) where the axis is parallel to the Earth’s gravitational force. Figure 3a shows the sensitivity of the accelerometer under gravitation. This result demonstrates that the accelerometer operates well and exhibits quite a linear performance even in the whole range from −90° to +90°. With the rotation, the resistance of the temperature sensor changes due to the shifting of the temperature profile in the cavity. The magnitude of the resistance change is measured as the sensitivity of the accelerometer under given conditions. The static sensitivity of the accelerometer is 360 μV·g^−1^ under a heater power of 50 mW.

The other method of measuring the sensitivity of the accelerometer, which is called dynamic sensitivity, is to intentionally apply acceleration to the accelerometer using a vibration shaker within a certain range of frequencies. Figure 3b shows that the accelerometer displays linear sensitivities with the range from 0 to 10 g at 30 Hz. In this case, the slope of the graph can be considered as the dynamic sensitivity of the accelerometer. We note that the dynamic sensitivity of the accelerometer is 277 μV·g^−1^ at an operating power of 50 mW. The static sensitivity is larger than the dynamic sensitivity under equal heating power.

**Figure 4:**
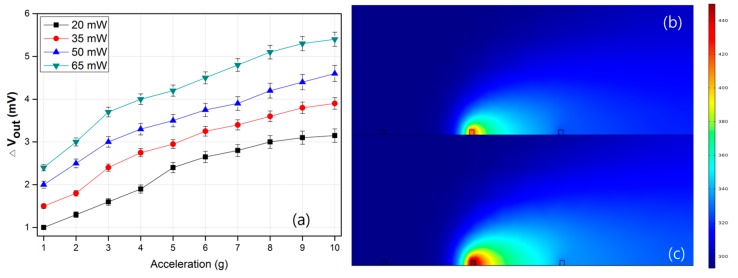
(**a**) The output voltage of the accelerometer as a function of acceleration with respect to the applied heater power and simulation results at a heater temperature of (**b**) 400 K and (**c**) 450 K (acceleration of 1 g and atmospheric pressure).

Figure 4a shows the effect of the heating power on the sensitivity of the accelerometer. The sensitivity of the accelerometer is shown to increase under a large heating power. As shown in Figure 4b,c, increasing the heating power can raise the temperature difference in the cavity of the accelerometer, simultaneously providing high sensitivity. A powered heater with high temperature contains a large amount of thermal energy compared to an unpowered heater or a heater with a low temperature. The law of conversion of energy states that the total energy of an isolated system remains constant. Energy can neither be created nor destroyed. However, it can be transformed from one form to another. In other words, powered heaters with high temperature can transfer a large amount of thermal energy to the working gas medium, which leads to high sensitivity of the thermal convection–based accelerometer [18]. In practical applications, however, we need to consider the power consumption and sensitivity of the accelerometer.

### 3.2. Effects of the Medium Type

Because the thermal convection–based accelerometer detects the temperature distribution profile of the gas medium that fills the cavity, cavity conditions such as the pressure, size, and gas medium are critical parameters that affect the sensitivity of the accelerometer.

**Figure 5:**
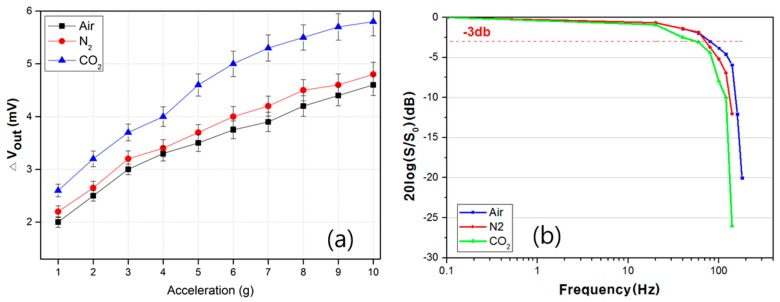
The output characteristics of the accelerometer as a function of the gas medium with respect to: (**a**) acceleration and (**b**) frequency.

The performance of the accelerometer filled with different gas media has been experimentally tested and analyzed. Figure 5a shows the effect of a gas medium on the sensitivity of the accelerometer with respect to different gas media, namely air, N_2_, and CO_2_. We can clearly observe that the accelerometer filled with CO_2_ shows the highest sensitivity, whereas the lowest sensitivity is observed in the accelerometer filled with air. The difference in the sensitivities under different gas media is due to the different properties, densities, and kinematic viscosities. As shown Table 1, CO_2_ has the largest density and smallest kinematic viscosity among these media, and its corresponding G_r_ value is the largest. In contrast, air has a small density and a large kinematic viscosity, thus yielding a low sensitivity. High viscosity makes a gas medium more resistant to gas flow, leading to lower sensitivity.

The frequency response of the accelerometers under different gas media has also been measured, and the results are shown in Figure 5b. The frequency response of the accelerometer filled with air gas media is nearly flat up to approximately 80 Hz. Beyond 80 Hz, the sensitivity substantially decreases as the frequency increases. When the acceleration is kept constant, the accelerometer moves a shorter distance at a higher frequency, which leads to a decrease in the sensitivity of the accelerometer [11]. Another reason is ascribed to the fact that higher frequencies induce a greater admixture of the hot and cold gas media surrounding the temperature sensors in the microchamber, thus degrading the sensitivity. Furthermore, the gas flow can change from a laminar to a turbulent flow at higher frequencies (normally > 70 Hz) [19].

On the other hand, the experiment results reveal that air can accurately detect the acceleration in frequency ranges of up to 80 Hz, whereas the CO_2_ can barely measure up to 60 Hz. The medium properties directly determine how rapidly the gases can move toward the temperature sensor. Gases with a lighter density and larger thermal diffusivity are advantageous as a working medium for a higher frequency response. A larger thermal diffusivity and a lighter density take less time to deliver the heated gas from the central microheater to the temperature sensor. From these factors, we can assume that the sensitivity of the accelerometer has a trade-off relationship with the frequency response.

### 3.3. Effects of the Gas Pressure

**Figure 6:**
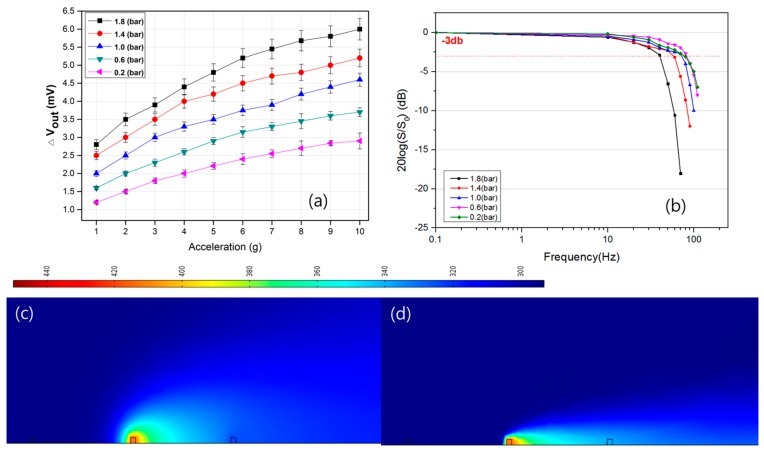
The output characteristics of the accelerometer as a function of pressure with respect to: (**a**) acceleration and (**b**) frequency. Simulation results at gas pressure of (**c**) 1 bar and (**d**) 1.8 bar (heater: 450 K).

The physical states of a gas medium in the cavity highly influence the sensitivity of the thermal accelerometer. A larger G_r_ number is beneficial for a sensitive thermal sensor. When the heater power, structural dimensions, and gas medium are given, the gas density is the only variable to be considered. The gas density ρ in Equation (1) corresponds to the ambient pressure. Thus, higher gas pressure increases the Gr number, improving the sensitivity of the thermal accelerometer. Figure 6a shows that better sensitivities are observed at higher gas pressure, as expected. Mailly et al. reported that the thermal boundary layer of the temperature profile decreases when the gas pressure (density) increases (the thermal boundary layer is defined as the distance in which the temperature rise is 10% of the heater temperature rise) [21]. As shown in the simulation results in Figure 6c,d, the reduced thermal boundary layer was observed at higher gas pressure. Although the thermal boundary layer is limited by the cavity size or volume of the accelerometer, according to this definition, the temperature sensor can detect higher temperatures at higher gas pressures without structural modification of the accelerometer. This feature is the special advantage of this sensor type because we can possibly achieve high sensitivity by packaging at higher pressure. In contrast, the proof-mass–type sensor requires modification of the structural parameters to improve the sensor performance.

On the other hand, an inferior frequency response is observed at higher air pressure, as shown in Figure 6b. As revealed by the expression of the thermal diffusivity, α = k/ρ·c, the density (ρ) of the gas is linearly proportional to the pressure, whereas the thermal conductivity (k) and specific heat (c) undergo very small variations even if the pressure increases [22]. These factors explain why the frequency response of the sensor decreases as its internal pressure increases.

### 3.4. Effects of the Cavity Volume

**Figure 7:**
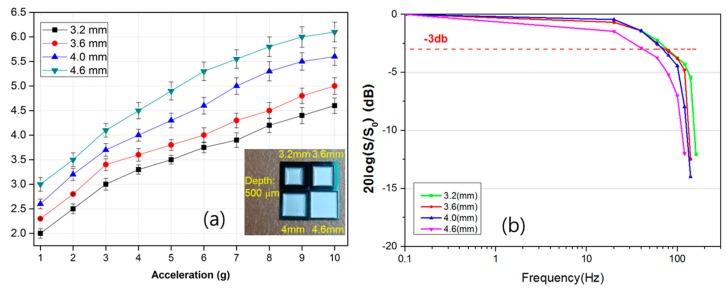
The output characteristics of the accelerometer as a function of the cavity volume with respect to: (**a**) acceleration and (**b**) frequency (inset: different cavity size).

The volume of the gas medium cavity also influences the sensitivity and frequency response of the sensor because it is related to the gas density and the heat quantity per unit volume of the sensor. The encapsulated microcavity is designed to accommodate various sizes to determine the optimum cavity volume. Figure 7a shows the output characteristics of the accelerometer as a function of the cavity size. The experiment reveals that the measurement results of the accelerometer show a higher sensitivity for a larger cavity because a smaller cavity retards the flow circulation [23]. Gas flow is restricted by a small cavity boundary, which substantially suppresses the flow action induced by thermal convection and reduces the sensitivity. Therefore, manufacturing using a large cavity size will increase the sensitivity of the accelerometer. These results are consistent with those of previously reported papers [15,16,17,18]. Figure 7b shows the frequency response of the proposed convective sensor with different cavity volumes. We find that a larger chamber has a lower frequency, which implies that a longer time is required for the temperature to reach equilibrium [12]. Thus, small sizes might be more desirable to enhance the frequency response. Based on this result, we can see that simultaneously improving both the sensitivity and frequency response by optimizing the gas medium, gas pressure, and cavity size is not possible. Therefore, in designing the thermal accelerometer, we need to consider the practical applications of the accelerometer.

## 4. Conclusions

An accelerometer based on convection was fabricated and tested in this study. Several characterizations derived through experimentation demonstrated that the thermal convection–based accelerometer exhibits better linearity, preferable frequency response, and higher sensitivity by optimizing various parameters. This study paid particular attention to the frequency response of the convection-based accelerometer based on thermal exchanges. The characteristics of the sensor have been experimentally investigated as a function of the gas medium, pressure, heater power, and cavity volume. The experiment results have shown that the frequency response can be extended by optimizing various factors, such as a smaller cavity volume, a lower gas pressure, and a gas medium with a large thermal diffusivity.

## Figures and Tables

**Table 1:** The gas medium properties at 50 °C [20].

	Density (kg/m^3^)	Specific Heat (kJ/kg·K)	Kinematic Viscosity (×10^−6^) (m^2^/s)	Thermal Diffusivity (×10^−4^) (m^2^/s)	Thermal Conductivity (W/m·K)
Air	1.092	1.007	19.6	0.248	0.02735
N_2_	1.0564	1.042	17.74	0.249	0.02746
CO_2_	1.6597	0.8666	9.71	0.129	0.01858
